# Multivoxel Pattern Analysis Reveals 3D Place Information in the Human Hippocampus

**DOI:** 10.1523/JNEUROSCI.2703-16.2017

**Published:** 2017-04-19

**Authors:** Misun Kim, Kate J. Jeffery, Eleanor A. Maguire

**Affiliations:** ^1^Wellcome Trust Centre for Neuroimaging, Institute of Neurology, University College London, London WC1N 3BG, United Kingdom, and; ^2^Institute of Behavioural Neuroscience, Division of Psychology and Language Sciences, University College London, London WC1E 6BT, United Kingdom

**Keywords:** 3D, hippocampus, isotropic, navigation, retrosplenial, virtual reality

## Abstract

The spatial world is three dimensional (3D) and humans and other animals move both horizontally and vertically within it. Extant neuroscientific studies have typically investigated spatial navigation on a horizontal 2D plane, leaving much unknown about how 3D spatial information is represented in the brain. Specifically, horizontal and vertical information may be encoded in the same or different neural structures with equal or unequal sensitivity. Here, we investigated these possibilities using fMRI while participants were passively moved within a 3D lattice structure as if riding a rollercoaster. Multivoxel pattern analysis was used to test for the existence of information relating to where and in which direction participants were heading in this virtual environment. Behaviorally, participants had similarly accurate memory for vertical and horizontal locations and the right anterior hippocampus (HC) expressed place information that was sensitive to changes along both horizontal and vertical axes. This is suggestive of isotropic 3D place encoding. In contrast, participants indicated their heading direction faster and more accurately when they were heading in a tilted-up or tilted-down direction. This direction information was expressed in the right retrosplenial cortex and posterior HC and was only sensitive to vertical pitch, which could reflect the importance of the vertical (gravity) axis as a reference frame. Overall, our findings extend previous knowledge of how we represent the spatial world and navigate within it by taking into account the important third dimension.

**SIGNIFICANCE STATEMENT** The spatial world is 3D. We can move horizontally across surfaces, but also vertically, going up slopes or stairs. Little is known about how the brain supports representations of 3D space. A key question is whether horizontal and vertical information is equally well represented. Here, we measured fMRI response patterns while participants moved within a virtual 3D environment and found that the anterior hippocampus (HC) expressed location information that was sensitive to the vertical and horizontal axes. In contrast, information about heading direction, found in retrosplenial cortex and posterior HC, favored the vertical axis, perhaps due to gravity effects. These findings provide new insights into how we represent our spatial 3D world and navigate within it.

## Introduction

The neural circuitry underlying spatial navigation is one of the most widely studied topics in neuroscience. The hippocampus (HC) and entorhinal (EC) and retrosplenial (RSC) cortices are key brain structures that have been implicated in building an internal map of the environment (for review, see Hartley et al., 2014). To date, however, most research has been conducted using simplified laboratory setups such as 2D flat arenas, yet humans and other animals live in a more complex 3D spatial world that includes undulating terrain, multilevel buildings, and open volumetric spaces. To build an accurate map of 3D space, the brain must have a system that encodes horizontal and vertical spatial information in an efficient manner. The navigation challenges faced by an animal when it navigates in 3D space are complicated by gravity. This imposes an energy constraint for moving along one axis, the vertical, so the vertical axis is distinguished from the other two horizontal axes in the world and perhaps in the brain.

Few studies have investigated the neural representation of 3D space and it remains unclear where and how vertical and horizontal information is encoded in the brain. Hayman et al. (2011) recorded place and grid cells in the HC and EC of rats moving on a vertical wall and helix staircase. In both apparatuses, place and grid cells expressed less information about the vertical axis, leading the investigators to propose that 3D space representation might be fundamentally planar (Jeffery et al., 2013). In contrast, most place cells recorded in CA1 of flying bats were equally sensitive to all three axes, suggesting isotropic representation of 3D space (Yartsev and Ulanovsky, 2013; for review, see Finkelstein et al., 2016; for a review of fish behavior in 3D, see Burt de Perera et al., 2016). Unlike place cells, which contained both vertical and horizontal information, the majority of head direction cells recorded in the presubiculum of crawling or flying bats contained either vertical or horizontal direction information alone (Finkelstein et al., 2015).

In humans, to the best of our knowledge, only a few behavioral (Vidal et al., 2004; Hölscher et al., 2006; Buechner et al., 2007; Barnett-Cowan et al., 2012) and neuroimaging studies have investigated navigation in 3D space. Two fMRI studies found that the HC was more engaged by horizontal than vertical motion (Indovina et al., 2013, 2016). In contrast, Zwergal et al. (2016) reported, using positron emission tomography (PET), a similar degree of hippocampal activation for navigation on a horizontal floor and across multiple floors of a building. However, the limitations imposed by the PET methodology, a between-groups design that contrasted overall activation between two navigation conditions, means that we still do not know whether information about specific vertical and horizontal locations is encoded in the HC and other navigationally relevant brain areas.

Therefore, in the present study, we investigated whether 3D location and direction information is represented in the human brain using an fMRI virtual navigation paradigm. Participants moved along flat, tilted-up or tilted-down pathways within a 3D lattice structure as if riding a rollercoaster and fMRI multivoxel pattern analysis was used to test for the presence of information pertaining to where and which direction a participant was heading in this virtual environment. The aim was to adjudicate between the following hypotheses: (1) vertical and horizontal information is similarly represented in a brain structure (isotropic encoding); (2) either vertical or horizontal information is represented with greater sensitivity in one or more brain regions (anisotropic encoding); or (3) vertical and horizontal information is represented in separate brain areas (2D planar encoding). Based on the vast navigation neuroscience literature, we focused on the HC, the EC, and RSC as priori regions of interest (ROIs).

## Materials and Methods

### 

#### Participants

Thirty-six healthy right-handed adults took part in the experiment (18 females, mean age 24.2 years, SD 4.25, range 19–34). All had normal or corrected-to-normal vision and gave informed written consent in accordance with the local research ethics committee.

#### Virtual reality environment

**Figure 1.**
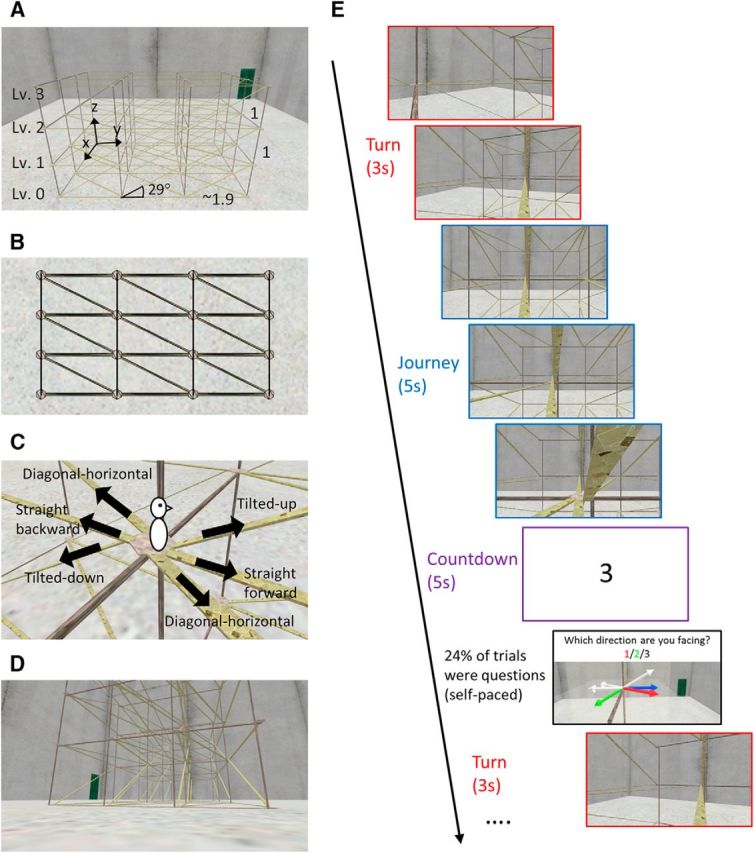
Experimental design. ***A***, Overview of the virtual environment. A 4 × 4 × 4 lattice structure was enclosed by tall concrete walls. One of the walls contained a green door as a unique landmark. The horizontal (*x*-axis) and vertical (*z*-axis) distance between two adjacent nodes was made identical to test the isotropy of 3D space encoding. The distance along one horizontal axis (*y*-axis) was 1.9 times longer than other distances because the vertical slope was designed to be walkable at 29°. ***B***, Overhead view of the lattice. ***C***, Close-up view of six pavements around a center node. From where a subject stood on a node, they could move along the pavements to one of six neighboring nodes: four on the same horizontal plane and two on different floors. ***D***, Example view of the lattice structure from a subject's perspective during the free exploration phase before scanning. ***E***, Example of a trial during scanning. Participants continuously moved from one node to another as if they were riding a rollercoaster. Each trial began with a turn at the node, followed by a linear movement on the pavement (journey) and then a countdown screen. In some trials, a question was presented before the next trial began.

The virtual environment was a lattice structure that conveyed the sense of an open and volumetric 3D space (Fig. 1*A*,*B*). It comprised 4 levels and each level contained 4 × 4 nodes that were linked to neighboring nodes by narrow pavements (horizontal or sloped) or wooden pillars. From where a subject stood on a node, they could move along the pavements to one of six neighboring nodes: four on the same horizontal plane and two on different floors. From an egocentric perspective, they could move straight forward or straight backward (in the latter case, they would turn 180° to approach the node behind them), diagonally on the same floor forward or backward, or up or down via a slope (Figs. 1*C*, 3*B*). The lattice was enclosed by tall concrete walls without a ceiling. All walls looked identical except that one contained a green door that acted as a unique landmark. During the experiment, the participants could only occasionally see the green door, so they could not use a simple landmark-matching strategy to know where they were; instead, they had to carefully keep track of their location throughout the experiment.

The horizontal (*x*-axis, see Fig. 1*A* for the axes conventions) and vertical (*z*-axis) distance between two adjacent nodes were made identical to test the isotropy of vertical and horizontal space representation in the human brain. The distance along the *y*-axis was set to be 1.9 times larger than the other two distances to make the slope of the pavement 29°. This angle was chosen to preserve ecological validity because this has been reported to be the steepest slope a human can walk up at a normal pace (Kinsella-Shaw et al., 1992).

The virtual environment was implemented using Unity 4.6 (Unity Technologies). A first-person perspective was used and the field-of-view was ±30° for the vertical axis and ±45.7° for horizontal axes. A snapshot of the 3D lattice as seen from a participant's perspective is shown in Figure 1*D*. During prescan training, the stimuli were rendered on a standard PC (Dell Optiplex 980) with an integrated graphic chipset and presented on a 20.1 inch LCD monitor (Dell 2007FP) with a screen resolution of 1600 × 900. The same PC and resolution were used during scanning. The stimuli were projected on the screen using an Epson EH-TW5900 projector at the back of the MRI scanner bore and participants saw the screen through a mirror attached to the head coil. The screen covered a field of view of ∼21° horizontally and ∼12° vertically.

#### Procedure

Each participant completed the experimental tasks in the following order: free exploration before scanning, one practice of the experimental task before scanning, the experimental task during scanning, and a postscan debriefing session.

##### Free exploration before scanning.

Having watched a short demonstration of the experimental task, participants explored the virtual environment freely using a keyboard in a testing room. During this self-paced exploratory period (mean duration 734 s, SD 300 s), participants volitionally moved along the pavements and visited all four floors of the lattice structure. The position and heading direction of participants were recorded every 0.1 s. Because the pavements linking each node were designed to be narrow, most participants “fell” down from the lattice at least once during the exploration. We welcomed this experience because it allowed participants to appreciate the height, maximizing the sense of 3D space. However, to prevent height-related anxiety from influencing the task, participants were told that they would move along a preprogrammed route during the scanning experiment without falling off. We later confirmed in the debriefing session that 94% of the participants were not at all anxious during the scanning experiment. Participants did not practice in advance the exact routes that would be experienced in the scanner because different pseudorandomized routes that were optimized for sampling each direction and place were used during scanning.

##### Scanning task.

In the scanner, participants moved along a preprogrammed route in the 3D lattice structure as if they were riding a rollercoaster (this was practiced before entering the scanner). This constrained-movement approach had advantages over unconstrained free exploration or the use of static picture stimuli. Compared with the latter, the rollercoaster experience provided participants with a strong sense of being in a 3D space (see debriefing results), thereby allowing a more ecological investigation of space representation in the human brain. Our approach also permitted us to control precisely the movement trajectory for every subject, which cannot be achieved if participants are freely exploring. We used movement sequences in which each of the inner eight nodes and directions of interest were sampled with similar frequency (see Fig. 3), allowing an unbiased and reliable estimation of 3D place and direction representations. The routes were presented in a randomized fashion across subjects. To ensure that subjects paid attention during the task, they were occasionally asked about their current position and direction (see below and Fig. 1*E* for a timeline of a trial).

A preprogrammed route during one scanning run was composed of 50 consecutive movements (trials) from one node to an adjacent node in the lattice. On each trial, the rollercoaster prepared to move at the initial node by turning toward the next node (“turn”). A constant angular velocity was applied during this 3 s turn and the instantaneous direction was a linear interpolation between the initial 3D direction vector and the next directional vector. After the turn, the rollercoaster underwent a linear movement along the pavement (“journey”) at a constant speed to the next node, which took 5 s. Participants' viewing angle, equivalent to their head direction, was parallel to the pavement. This meant that when they were moving up by 29°, head pitch was also 29°. Having arrived at the end node, the virtual environment was temporarily hidden by a white countdown screen for 5 s (“countdown”). In the majority of trials (76%), the next trial started right after the countdown. In 24% of the trials, a question was presented before the next trial and the subject indicated their current position or direction on a map using an MR-compatible keypad with the right hand. These occasional questions were included to maintain participants' attention and to compare the behavioral sensitivity of encoding the vertical and horizontal dimensions (see the behavioral analysis section). The question period also helped participants to maintain the correct sense of direction throughout the experiment because, when participants answered incorrectly, the correct place or direction was shown on the screen. In total, one run of 50 consecutive trials lasted ∼13 min. Participants completed four runs with a short break between each run, making the total functional scanning time ∼50 min.

##### Postscan debriefing session.

After scanning, participants were asked about how much they felt immersed in the virtual environment with the following options: “I felt like I was really there”; “I occasionally thought about the environment as being on a computer screen, but overall the environment was convincing and I felt I was moving straight, up or down”; or “I was often distracted by the feeling that I was not in a real environment.” They also reported whether the height made them anxious or nervous during the scanning task with three options: “not at all,” “somewhat,” “very.”

Importantly, participants were asked (without prior notice) to estimate the length and angle of the virtual environment in terms of meters and degrees based on their experience of navigating within it. Although the vertical and horizontal distance between two nodes were made equivalent, participants' subjective perception of distance or direction could be different from the true physical distances due to the horizontal–vertical visual illusion (Avery and Day, 1969) and this might influence the neural encoding of the vertical and horizontal dimensions. For instance, the neural representation of the straight heading direction and the vertically 29° tilted direction could be more dissimilar than the straight direction and horizontally 29° tilted direction if participants overestimated the vertical slope compared with the horizontal angle. In addition to the quantitative estimate of vertical/horizontal size, we also asked about participants' qualitative impression of the size of the whole environment (options: small/medium/large) because spatial scale-dependent representation has been associated with the HC (Evensmoen et al., 2015).

#### Behavioral analysis

##### Performance during the scanning task.

**Figure 2.**
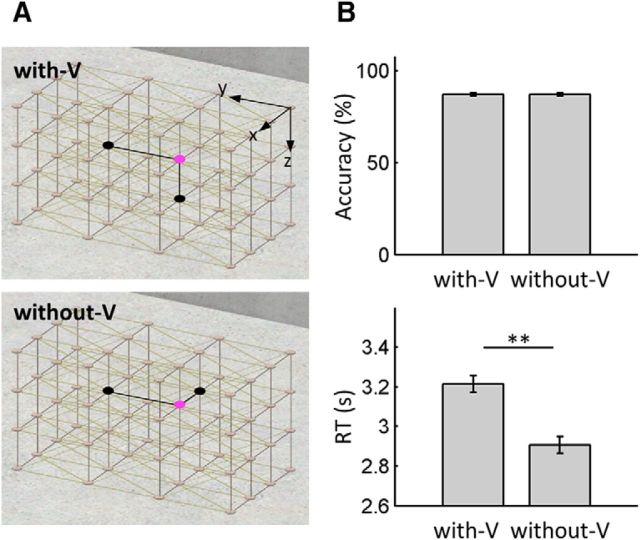
Behavioral analysis of vertical and horizontal place encoding. ***A***, Positions of the distractors in the place questions. For simplicity of explanation, a correct node here is shown in pink and the two distractors are shown in black in orthogonal projections of the 3D lattice; in the actual experiment, red, green, and blue were used to denote the three choices in a first person perspective as in Figure 1*A*. In the with-V condition, one distractor was above or below the correct node and the other distractors were adjacent to the correct position on the same horizontal plane. In the without-V condition, both distractors were on the same floor as the correct location. ***B***, Accuracy and RT for the different place questions. Accuracy did not differ between the conditions (top), but RT was significantly shorter in the without-V condition (bottom), indicating that distinguishing the vertical position took longer than locating one's position within a horizontal plane. Error bars are SEM adjusted for a within-subjects design (Morey, 2008). **p < 0.01.

Both place and direction questions were three-alternative forced choice, meaning that chance accuracy was 33%. In the place question, the positions of the two distractors varied systematically, enabling us to compare the behavioral sensitivity of horizontal and vertical encoding (Fig. 2*A*). In the “with-V” condition, one distractor was above or below the correct position (vertical distractor) and the other distractor was on the same floor adjacent to the correct position. In the “without-V” condition, all three choices were on the same horizontal plane such that one distractor was adjacent to the correct position along the short horizontal axis and the other along the long horizontal axis. If the vertical and horizontal axes were equally well encoded, performance for both conditions should be similar. Conversely, if the vertical axis was poorly encoded relative to the other two horizontal axes such that the participants were more confused by a distractor above or below the true position, then performance for the with-V condition would be worse than for the without-V condition, which did not involve a vertical distractor. We compared the response time (RT) and accuracy of these conditions using a paired *t* test. All statistical tests were computed in MATLAB or SPSS and data are presented as mean ± 1 SD unless otherwise specified.

**Figure 3.**
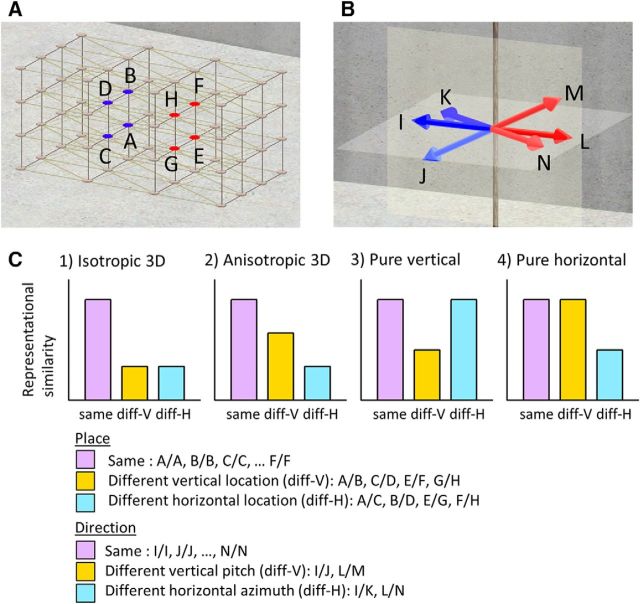
Place and direction encoding hypotheses. ***A***, Places of interest. The inner eight nodes on the middle two floors were used because the ground level and the top floor were visually distinctive. ***B***, There were six heading directions. ***C***, Hypotheses: (1) The isotropic 3D encoding hypothesis predicts that the vertical and horizontal axes are symmetrically encoded. Therefore, two places along the vertical axes (diff-V, e.g., A and B) are equally distinguishable as the two points along the horizontal axes (diff-H, e.g., A and C), resulting in equal representational similarity for diff-V and diff-H conditions that are smaller than the same location condition (same, e.g., A and A). In the case of direction encoding, two directions that have different vertical pitch components (diff-V, e.g., L and M) would have similar pattern similarity as two directions that have different horizontal azimuth components (diff-H, e.g., L and N). (2) The anisotropic, horizontal-weighted hypothesis predicts higher pattern similarity for diff-V than diff-H because the neural response is less sensitive to the vertical change than to the horizontal change. (3) The pure vertical encoding hypothesis predicts that, as long as the vertical coordinate is the same, the neural pattern will be equivalent even if the horizontal coordinate is different and therefore diff-H is comparable to the same. (4) The pure horizontal encoding hypothesis predicts the opposite, that diff-V is comparable to the same.

The direction questions were included to motivate the participants to concentrate on both place and direction and there was no variation in the distractors. Rather, we compared the RT and accuracy when the correct direction had a nonzero vertical pitch component (direction J/M in Fig. 3*B*) and when the pitch of the correct direction was zero (direction I/K/L/N in Fig. 3*B*) to test whether vertical pitch was more distinguishable.

##### Postscan debriefing session.

We counted the number of responses for each option in the multiple choice debriefing (i.e., participants' engagement in the virtual environment, emotional state and qualitatively perceived size of the environment). A quantitative size estimate of the vertical and horizontal dimensions was analyzed using a *t* test. A ratio of the perceived vertical and horizontal distance and angle was tested against a true ratio of 1 using a 1-sample *t* test.

#### Scanning and image processing

T2*-weighted echo planar images (EPIs) were acquired using a 3T Siemens Trio scanner with a 32-channel head coil. Scanning parameters optimized for reducing susceptibility-induced signal loss in areas near the orbitofrontal cortex and medial temporal lobe were used: 48 transverse slices angled at −30°, TR = 3.36 s, TE = 30 ms, resolution = 3 × 3 × 3 mm, matrix size = 64 × 74, *z*-shim gradient moment of −0.4 mT/ms (Weiskopf et al., 2006). Field maps were acquired with the standard manufacturer's double echo gradient echo field map sequence (short TE = 10 ms, long TE = 12.46 ms, 64 axial slices with 2 mm thickness and 1 mm gap yielding whole-brain coverage; in-plane resolution 3 × 3 mm). After the functional scans, a 3D MDEFT structural scan was obtained with 1 mm isotropic resolution.

Data were preprocessed using SPM12 (www.fil.ion.ucl.ac.uk/spm). The first five volumes from each functional session were discarded to allow for T1 equilibration effects. The remaining functional images were realigned to the first volume of each run and geometric distortion was corrected by the SPM unwarp function using the field map. Each participant's anatomical image was then coregistered to the distortion corrected mean functional images. Functional images were normalized to MNI space and left unsmoothed for multivoxel pattern analysis to preserve the fine-scale activity patterns.

#### Anatomical ROIs

**Figure 4.**
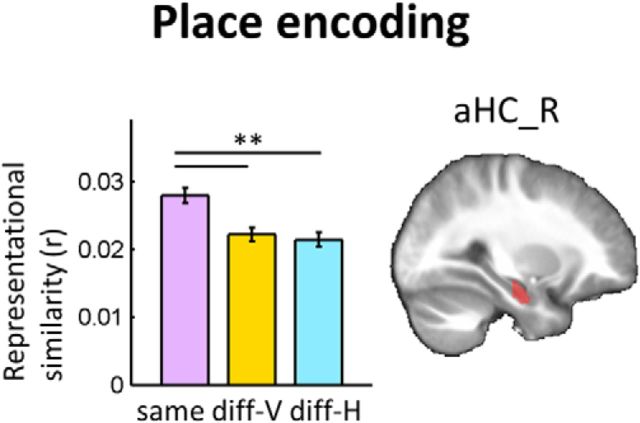
Place encoding results. The right aHC (aHC_R) contained significant place information. Locations along the vertical axis (diff-V) and locations along the horizontal axis (diff-H) were equally distinguishable (same > diff-V or diff-H), suggesting an isotropic 3D representation. The anatomical ROI is overlaid on the group average structural MRI scan. Error bars are SE of mean adjusted for a within-subjects design (Morey, 2008). **p < 0.01, *post hoc* Bonferroni-corrected.

**Figure 5.**
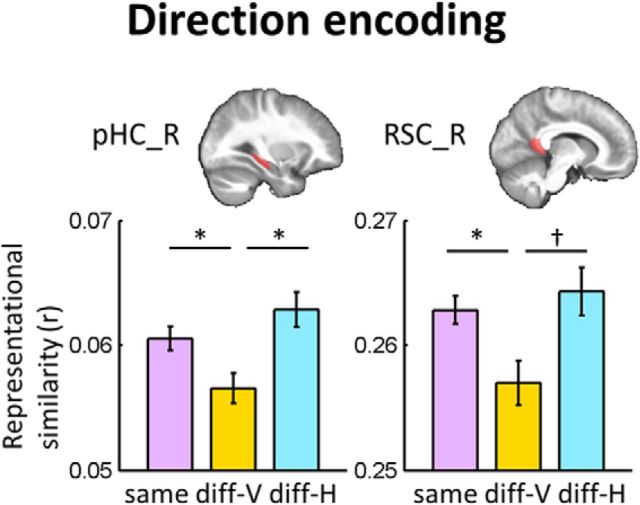
Direction encoding results. The right pHC (pHC_R) and right RSC (RSC_R) ROIs contained significant direction information. In both regions, different vertical directions were distinct (same > diff-V) but the horizontal directions were not (same ≈ diff-H). The anatomical ROI is overlaid on the group average structural MRI scan. Error bars are SE of mean adjusted for a within-subjects design (Morey, 2008). *p < 0.05, †*p* = 0.12, *post hoc* Bonferroni-corrected.

We defined three anatomical ROIs for areas known to contain cells sensitive to spatial information: the HC, RSC, and EC. Each ROI was manually delineated on the group average structural MRI scan (1 × 1 × 1 mm) using ITK-SNAP (www.itksnap.org) and then resampled to the functional scans (3 × 3 × 3 mm). The HC was divided into anterior (aHC) and posterior (pHC) ROIs given the literature showing anatomical and functional variation along its long axis (Poppenk et al., 2013). A coronal coordinate (*y* = −19 mm) that approximates the position of uncal apex on our group average structural scan was used to divide the HC into the aHC and pHC sections. The RSC included Brodmann areas 29–30 (Vann et al., 2009). The EC ROI was defined following the protocol in Pruessner et al. (2002). We defined the caudal end of EC as 2 mm posterior to the uncal apex (*y* = −21 mm) following this protocol, but note that some studies have used a more posteriorly extended definition of EC (Chadwick et al., 2015). The EC is challenging for fMRI researchers due to substantial signal loss induced by susceptibility artifact in this region. We assessed the temporal signal-to-noise ratio (tSNR) defined as the mean of the normalized EPI time series divided by the SD in every voxel of our ROIs. As expected, tSNR was much lower in EC (19.8 ± 6.6) compared with the whole HC (61.1 ± 8.9) and RSC (56.2 ± 9.9), implying that EC was disadvantaged in expressing its function. Anatomical localization of the ROIs can be seen in Figures 4 and 5 The number of functional voxels within each ROI (L = left, R = right) was as follows: aHC_L, 54; aHC_R, 61; pHC_L, 98; pHC_R, 91; RSC_L, 158; RSC_R, 135; EC_L, 47; and EC_R, 49.

#### Multivoxel pattern analysis

##### 3D space encoding hypotheses.

To adjudicate between the different 3D space encoding hypotheses, isotropic 3D, anisotropic 3D, or planar, we compared the amount of vertical and horizontal spatial information in each ROI using multivoxel pattern similarity analysis (Haxby et al., 2001; Kriegeskorte et al., 2008). Our assumption was that, if both the vertical and horizontal dimensions were encoded with equal sensitivity (Fig. 3*C*, isotropic), then the neural representation of the two points along the vertical axes should be as distinguishable as those of the two points along the horizontal axes given that the distances between these two points are equivalent. Therefore, in Figure 3*A*, fMRI multivoxel pattern similarity between place A and B (different vertical, “diff-V”) would be comparable to the similarity between place A and C (different horizontal, “diff-H”) and, obviously, both should be lower than the within-place (A and A) pattern similarity (“same”). If the vertical axis is poorly encoded compared with the horizontal axis (Fig. 3*C*, anisotropic, horizontal weighted), then the two places along the vertical axes would be less distinguishable than the two places along the horizontal axes. Therefore, the pattern similarity between place A and B (diff-V) would be larger than the similarity between A and C (diff-H). In contrast, if the horizontal axis is encoded with low sensitivity (vertical weighted, not shown), then the pattern similarity of the diff-H condition would be larger than that of the diff-V condition. In the extreme case when only the horizontal (or vertical) dimension is encoded (Fig. 3*C*, pure horizontal or pure vertical), the neural representation of two places that share the same horizontal (or vertical) coordinates would be completely indistinguishable, so the pattern similarity for the two positions along the vertical axis, the diff-V condition (or the two positions along the horizontal axis, diff-H condition), would be comparable to same place condition. If neither horizontal nor vertical information is encoded, then neural responses to each location will be random and inconsistent, so there would be no systematic differences between the same, diff-V, and diff-H conditions. An analogous analysis was used to test for the existence and quantity of horizontal (azimuth) and vertical (pitch) direction information in the ROIs.

##### Analysis protocol.

The first step for the multivoxel pattern analysis was to estimate neural representations (multivoxel patterns) for each place and direction in the virtual 3D lattice structure. Although there were four levels in the virtual environment, only the inner eight nodes on the middle two floors were used for the analysis because the ground level and the top floor were quite distinctive in physical appearance (Fig. 3*A*). To increase the number of visits to these inner nodes, the rollercoaster moved between these inner nodes on 76% of the trials. Therefore, the four nodes marked in blue were usually approached from three directions marked in blue and the other four nodes marked in red were approached from the three directions in red (Fig. 3*A*,*B*). We estimated the unique multivoxel pattern activity for each place × direction pair (8 × 3 = 24) for each scanning session and each participant using the SPM general linear model (GLM). The GLM contained 24 place × direction regressors that modeled the journey + countdown period of 10 s for each of the four scanning sessions. The whole journey + countdown period was used because the participants reported that they thought about where they were moving from the beginning of the journey period and they had to maintain this spatial information until the end of countdown. In addition to the 24 regressors of interest for each session, nuisance regressors were included in the GLM; one for modeling the trials when the participants visited outside the inner eight nodes, two regressors for modeling the occasional place and direction question periods, six regressors for head motion realignment, and a constant regressor for each scanning session to account for mean signal variation. In summary, the resulting *t*-statistics for each voxel in the ROIs were the estimates of multivoxel activations when the subjects were facing those directions and moving toward and standing in those locations, with the hemodynamic delay being taken into account. The second step was to calculate the similarity of multivoxel patterns for each place and direction combination using Pearson's correlation coefficient and to compare the similarity with the 3D encoding hypotheses described in Figure 3.

Because the four nodes shown in blue and the four nodes shown in red in Figure 3*A* were approached by three different directions, as described above, we restricted the analysis to within either blue or red nodes to control fully the direction and place factors and then averaged the two similarity matrices later. As a result, a 12 × 12 pairwise correlation matrix was created for each subject. Importantly, we cross-validated the similarity measure across runs to ensure the independence of each dataset and to estimate a nonbiased similarity measure (e.g., the similarity between place A-direction 1 and place B-direction 2 was the mean of the correlation between the place A-direction 1 in run 1 and place B-direction 2 in run 2 and the correlation between run1 and run3 between run 1 and run 4, etc.) Each pairwise similarity measure was then grouped into three categories: same (e.g., A and A), different vertical (diff-V, e.g., A and B), and different horizontal (diff-H, e.g., A and C) (Fig. 3*C*).

For the place encoding analysis, the pairs of place × direction combinations that shared the same direction were excluded to control for the direction factor. By excluding the same direction pair, the neural representation similarity between the same place cannot be due to mere visual identity. Rather, if the neural representation is more similar for the same place compared with another place, then it can be interpreted as evidence for place encoding that is generalizable across different directions and different scanning sessions. For the direction encoding analysis, the same place pairs were excluded to test for the existence of direction information that was independent of place.

Finally, the mean pattern similarity for each of the three categories (same, diff-V, and diff-H) were compared at the group level with a one-way repeated-measures ANOVA and *post hoc t* test with Bonferroni correction. Normality of the data was confirmed by the Shapiro–Wilk test. Greenhouse–Geisser-corrected *p-*values are reported when sphericity assumptions were violated (Mauchly's test). We report one sided *p-*values for comparisons between same and diff-V or same and diff-H because the pattern similarity for the same spatial condition should be higher than those for a different condition if place or direction information is present. We then plotted the bar graphs of this mean pattern similarity so that they could be easily compared with our 3D place and direction encoding hypotheses that are shown in Figure 3*C*.

##### Control analysis: visual texture similarity.

We designed the virtual environment with a limited palette of colors and textures and only one salient landmark to minimize the influence of visual inputs when investigating place or direction information. Nevertheless, visual input differed second to second because participants solely relied on the optical inputs to track their position and direction. The floor in particular was salient because it provided an unambiguous and reliable sense of 3D space. Therefore, we applied a slightly brighter color to the floor and the proportion of the floor included in the field of view varied depending on whether the participants were heading straight or up/down. To determine whether the place or direction information found in the brain in the main analysis was explained by these visual differences, we conducted a control analysis that used partial correlations to compare the neural similarity data with the place or direction encoding hypotheses (Fig. 3) while controlling for this visual component.

We measured pairwise visual similarity between each place and direction using a simple visual texture model (Renninger and Malik, 2004) in the same way as neural pattern similarity was calculated (e.g., visual similarity between place A-direction 1 and place A-direction 2, the similarity between place C-direction 1 and place D-direction 2). Images captured at every half a second during the 5 s journey period of each place × direction combination were averaged and entered into the texture model. The model applied Gabor filters of varying orientation and size to extract the common textures and then a distribution of textures across the different pairs of images was compared using the χ^2^ distribution. The χ^2^ distance metric was converted to a similarity measure by subtracting each χ^2^ value from the maximum χ^2^ value. Therefore, we had “observed neural similarity” and “visual similarity” variables for each subject. Next, we created “predicted neural similarity” variables based on the isotropic place encoding or pure vertical direction encoding hypotheses (Fig. 3). The isotropic place encoding model predicts high neural similarity for the same place (e.g., place A-direction 1 and place A-direction 2) and low similarity for different places (e.g., place A-direction 1 and place B-direction 2); therefore a value of 1 was assigned for the pairwise similarity in the “same” condition and 0 was assigned for the “diff-V” and “diff-H” conditions. As long as the rank order is preserved, any number can be assigned. The pure vertical direction encoding model was assigned 1 for “same” and “diff-H” and 0 for “diff-V.” We then calculated the partial Spearman correlation between the “observed neural similarity” and “predicted neural similarity” while controlling the “visual similarity” for each subject. If this partial correlation is significantly above zero across subjects, it would be evidence of place or direction encoding in the neural data that is not fully accounted for by low-level visual features. This partial correlation approach is similar to that used by Carlin et al. (2011). We used a one-sided *t* test to determine significance after confirming the normality of the data using the Shapiro–Wilk test.

## Results

### Behavioral results

#### Performance during scanning

Overall, participants correctly kept track of their position and direction within the 3D lattice during the virtual rollercoaster ride (place question accuracy = 86.6 ± 12.3%, direction question accuracy = 93.4 ± 8.6%, chance level = 33.3%). The place questions were divided into two categories depending on the existence of a vertical distractor (Fig. 2*A*). Accuracy did not differ between the categories (*t*_(35)_ = 0.0, *p* = 1.0; Fig. 2*B*), but RT differed significantly. Participants responded faster when there was no distractor along the vertical axis (without-V, mean = 2.91 ± 0.71 s) compared with when a vertical distractor was present (with-V, mean = 3.21 ± 0.84 s, *t*_(35)_ = 3.7, *p* < 0.001). These results imply that the participants precisely identified themselves within a horizontal plane and the process of distinguishing the vertical coordinate (“Am I on the first floor or second floor?”) slowed down the response slightly without affecting accuracy.

In contrast, for the direction question, the presence of a slope speeded up responses. RT when the facing direction was tilted up or tilted down (mean = 2.26 ± 0.57 s) was significantly shorter than when the facing direction was on a horizontal plane (mean = 2.69 ± 0.63 s; *t*_(35)_ = −6.3, *p* < 0.001). Accuracy was higher in the vertical question trials (mean = 97.7 ± 4.9%) compared with the nonvertical trials (mean = 91.1 ± 12.6%; *t*_(35)_ = 3.3, *p* = 0.003). This facilitation is consistent with previous findings in humans and rats in which the slope of a maze facilitated spatial memory (Grobéty and Schenk, 1992; Steck et al., 2003).

#### Postscan debriefing session

When asked about their engagement with the task, 19% of participants chose the option “I felt like I was really there” and 69% chose “I occasionally thought about the environment as being on a computer screen, but overall the environment was convincing and I felt I was moving straight, up or down,” implying that our virtual environment provided an effective, if not complete, sense of being in 3D space. As noted above, we also confirmed that height-related anxiety was not the confounding factor because 94% of participants reported being “not at all anxious” during the scanning experiment.

Testing of the perception of the environment's size revealed qualitatively that the majority of subjects regarded the overall size of the virtual environment as medium or large (large: 31%, medium: 58%) and only 11% of the subjects reported it as small. Quantitatively, the ratio of vertical and horizontal distance estimates was not significantly different from the true ratio of 1 (mean ratio = 1.01 ± 0.27; *t*_(35)_ = 0.2, *p* = 0.8). This result is suggestive of unbiased, isotropic 3D space perception in our virtual environment. However, the participants estimated the angle between the slope and the horizontal plane as being significantly larger than the angle between two pavements on the horizontal plane (mean ratio = 1.15 ± 0.31, *t*_(35)_ = 2.8, *p* = 0.008) even though basic geometry would imply that the two angles should be identical given that the vertical and horizontal distances are equal. Our finding is consistent with the literature on human observers' tendency to overestimate the steepness of a slope (Proffitt et al., 1995) and vertical pointing in a 3D building (Brandt et al., 2015). The symmetric distance perception and asymmetric angle perception raised the question of whether the brain would encode vertical/horizontal place and direction symmetrically or asymmetrically.

### Multivoxel pattern analysis

#### Place encoding

Among our ROIs, the right aHC showed evidence of significant place information (*F*_(2,70)_ = 7.6, *p* < 0.001; Fig. 4). In the HC, both vertically displaced locations (diff-V) and horizontally displaced locations (diff-H) were significantly distinguishable from the same locations [normalized 95% confidence intervals (CIs) of the pattern similarity were as follows: same = 0.026–0.03, diff-V = 0.02–0.025, diff-H = 0.019–0.023; *post hoc* pairwise comparison, same > diff-V, *p* = 0.006, Cohen's d = 0.52; same > diff-H, *p* = 0.002, Cohen's d = 0.61, Bonferroni corrected). fMRI pattern similarity of vertically displaced locations (diff-V) and horizontally displaced locations (diff-H) were not significantly different from each other (diff-V versus diff-H, *p* = 1.0, Cohen's d = 0.12). Although the absence of significant difference between the vertical and horizontal place encoding is not direct evidence of equivalence between the two, the largely overlapping 95% CIs of diff-V and diff-H and our prior encoding hypotheses suggest that this finding best fits with the isotropic 3D place encoding hypothesis in which the horizontal and vertical dimensions are encoded with similar sensitivity (Fig. 3*C*, isotropic 3D). The right EC ROI showed a trend for pure vertical encoding (*F*_(2,70)_ = 2.3, *p* = 0.1, same > diff-V, *p* = 0.07). No other ROIs showed either vertical or horizontal place information. The absolute fMRI pattern similarity value of ∼0.02–0.03 may appear to be low, but given that neural signals are highly variable and the similarity was calculated across different scanning sessions to ensure complete independence of the datasets (while also controlling the direction factor for place and the place factor for direction), small values are to be expected. Indeed, these values are perfectly consistent with extant studies using this approach (Hsieh et al., 2014; Schapiro et al., 2016; Chadwick et al., 2015; Schuck et al., 2016; Hsieh and Ranganath, 2015). More importantly, the absolute similarity value within a single condition has very little meaning and the existence of place information should be tested by the difference in pattern similarity value between the conditions. Cohen's d, as reported above, provided an additional estimate of effect sizes. A supplementary control analysis confirmed that this isotropic place encoding in the aHC pertained after controlling for low-level visual features (*t*_(35)_ = 3.5, *p* = 0.0006).

#### Direction encoding

The direction encoding analysis revealed quite different results from the place encoding results. The right RSC and right pHC (pHC_R) expressed significant direction information (RSC_R, *F*_(2,70)_ = 3.8, *p* = 0.04; pHC_R, *F*_(2,70)_ = 4.8, *p* = 0.01; Fig. 5), whereas no other ROIs did so. In both RSC_R and pHC_R, different vertical directions (diff-V) were distinguishable (normalized 95% CIs of the pattern similarity were as follows: same 0.261–0.265, diff-V = 0.254–0.261, diff-H = 0.261–0.268; *post hoc* pairwise comparison, same > diff-V, *p* = 0.02, Cohen's d = 0.44 for RSC_R; 95% CIs were as follows: same = 0.059–0.062, diff-V = 0.054–0.059, diff-H = 0.060–0.066; same > diff-V, *p* = 0.03, Cohen's d = 0.42 for pHC_R), whereas the different horizontal directions (diff-H) were not. This result suggests a pure vertical encoding scheme in the right RSC and right pHC and this is consistent with previous animal studies finding that head direction cells that were only sensitive to the vertical pitch (Stackman and Taube, 1998; Finkelstein et al., 2015). This vertical direction encoding in the RSC and pHC cannot be explained by low-level visual features. A partial correlation analysis revealed significant vertical direction encoding after controlling for the visual texture similarity (pHC_R: *t*_(35)_ = 2.17, *p* = 0.02; RSC: *t*_(35)_ = 1.9, *p* = 0.03).

## Discussion

Here, we investigated the neural representation of 3D spatial information in the human brain using a combination of behavioral testing and fMRI multivoxel pattern analysis. There were three main results. First, behaviorally, participants had similarly accurate memory for vertical and horizontal locations, whereas vertical tilt facilitated performance on the direction judgment task. Second, we found that the right aHC contained place information that was sensitive to both horizontal and vertical axes. Finally, vertical directional information was found in the right pHC and RSC.

The HC is known for its role in encoding an animal's location (O'Keefe and Dostrovsky, 1971; Ekstrom et al., 2003; Hassabis et al., 2009; Sulpizio et al., 2014) and our findings extend previous knowledge by taking into account the third spatial dimension. Together with the behavioral findings of high accuracy regardless of the presence of a vertical or horizontal distractor and the symmetrically perceived length of the 3D environment, multivoxel representational similarity in the right aHC supports an isotropic 3D space encoding hypothesis and is evidence against the anisotropic planar encoding hypothesis (Hayman et al., 2011; Jeffery et al., 2013).

However, we do not claim that 3D space representation is unconditionally symmetrical in humans; rather, we believe that the neural representation of 3D space is flexible and dependent on various factors. First, the shape of the environment strongly influences the neural representation of space. It is well known that place cells show a repeating firing pattern when the environment comprises multiple recurring compartments (Nitz, 2011; Spiers et al., 2015) and 3D space is often divided into multiple horizontal segments (e.g., a multilevel building). Therefore, an apparent lack of vertical information in Hayman et al.'s (2011) rat study could be due to the repeating nature of the staircase apparatus along the vertical axis. In contrast, our 3D lattice environment was discretized into both vertical and horizontal axes with the same distance physically and perceptually and, in this circumstance, both axes were encoded with equal sensitivity. This could also be the reason for the isotropic 3D place fields observed in bats flying in open symmetrical space (Yartsev and Ulanovsky, 2013). A future study could test whether the human HC has less sensitive vertical encoding in an asymmetrical environment.

Behavioral demands and the mode of exploration can also affect 3D maps (Finkelstein et al., 2016). A place cell's response can be modulated by reward and attention (Markus et al., 1995; Hölscher et al., 2003) and it is possible that a place cell adapts to encode and remember the space better when it is behaviorally relevant. Unlike our participants, who were explicitly asked to encode both vertical and horizontal coordinates, most animal studies do not impose such a requirement. We suspect that place cells in rats could show an isotropic firing pattern if they were explicitly required to distinguish every location in 3D space. A recent behavioral study showed that rats were able to learn a 3D maze as well as a 2D maze, at least over a short timescale (Wilson et al., 2015). Conversely, the vertical and horizontal axes might be encoded differentially in humans when explicit spatial awareness is absent or a more demanding goal-oriented navigation task is used. Participants were passively moved here for optimal sampling of 3D locations and directions. A more ecological approach allowing free movement would be interesting for a future study.

Our other result concerned the encoding of vertical and horizontal directions. At the behavioral level, participants indicated their heading direction faster and more accurately when they were facing up or down and the vertical angle was overestimated to a greater extent than the horizontal angle. Our findings fits with the idea that the gravity (vertical) axis is a reference direction (Barnett-Cowan and Bülthoff, 2013). Knowing one's direction relative to the gravity axis is essential for maintaining the stability of body posture and all animals have a tendency to maintain an upright head posture. Physical gravity did not play a part in the current experiment because subjects were in a supine position in the MRI scanner. However, the vertical axis can be defined, not just by gravity, but also by visual, vestibular, and body orientation cues. There is extant psychological literature that has investigated the subjective and perceptual “upright.” For instance, Dyde et al. (2006) reported that orientation of the visual background scene is the dominant factor for judging an object's uprightness when subjects were tested in a supine position. We believe that the visually conveyed vertical axis in our virtual environment was a reasonable proxy for the gravity vertical axis in real life and that the experience of “falling” during the prescan free exploration also supported this analogy. At the neural level, the right pHC and RSC showed only vertical direction information. This might reflect potential head direction cells that are only sensitive to vertical pitch similar to those found in animals (Stackman and Taube, 1998; Finkelstein et al., 2015). Our direction encoding result remained significant after controlling for visual texture similarity, although we acknowledge that view and head direction, in particular for the vertical component, were not perfectly orthogonal. It is possible that the RSC and pHC findings may also be related to view encoding. RSC has connections to many cortical and subcortical regions that map space in different reference frames, including the HC, posterior parietal cortex, and thalamic nuclei (Vann et al., 2009). It encodes not only head direction but also turning behavior (Alexander and Nitz, 2015), place and view (Vass and Epstein, 2013; Marchette et al., 2014), and stable landmarks (Auger et al., 2012, 2015). Future work should seek to disentangle these factors and isolate 3D head direction information.

Our finding of different types of spatial information (place vs vertical direction) in the anterior and posterior HC is consistent with other evidence of functional variation along the longitudinal axis. Based on evidence from animal electrophysiology (Kjelstrup et al., 2008), lesion studies (McTighe et al., 2009), and neuroimaging (Evensmoen et al., 2015), it was proposed that the aHC may encode a large-scale or generalizable representation of the environment, whereas the pHC may encode a fine-scale and local representation (Poppenk et al., 2013; Zeidman and Maguire, 2016). In our experiment, the lattice structure eliminated the demand for fine-scale encoding of locations and most subjects perceived the size of the environment as medium or large rather than small. Therefore, the aHC may have been suitable for representing this location information independent of the direction. In contrast, the pHC could be associated with the vertical direction because the detail of a view was more distinguishable when participants were heading up or down. Posterior HC is connected to the parahippocampal and retrosplenial regions that are known for scene processing (Kobayashi and Amaral, 2003; Blessing et al., 2016) and are activated during scene discrimination tasks (Lee et al., 2008).

In conclusion, our experiment, one of the first investigations of 3D spatial representation in the human brain, opens up intriguing questions for future research. First, how does the human brain encode a continuous 3D space? Here, we used a discrete lattice environment, but the neural encoding of space and behavioral strategies could be different in a continuous environment. For instance, participants could encode their locations and direction in the lattice using a categorical method such as “second floor, upper left corner,” whereas a metric map is needed in an open environment such as “5.7 m from the floor,” “8° latitude, 11° longitude.” A potential subtle difference in sensitivity to the horizontal and vertical axes that was not detected in the current lattice structure environment might be revealed in a continuous environment. Second, is there lateralization of spatial encoding in the brain? We observed spatial information predominantly in the right hemisphere. Lateralized hippocampal function has been reported in patient studies (Maguire et al., 1996; Stepankova et al., 2004) and functional neuroimaging (Vass and Epstein, 2013; Baker et al., 2015; Schapiro et al., 2016), but there is as yet no clear explanation of possible laterality effects. Third, how can we investigate the brain mechanisms of 3D navigation with multisensory cues? Although humans can perceive 3D space from visual input alone and the current neuroimaging methodology constrains the use of other sensory cues, vestibular sensations are crucial for detecting one's heading relative to gravity (Angelaki et al., 2009) and there is some evidence for asymmetry in encoding vertical and horizontal dimensions in the vestibular system (Fernandez and Goldberg, 1971). Prescan training using a virtual reality headset that engages the vestibular system may improve the investigation of neural representations in future studies (Shine et al., 2016). Last, how 3D space is represented at the individual neuron level still remains a fundamental question. Vertical and horizontal location information could be equally well represented in fMRI multivoxel response patterns because each neuron has a symmetrical 3D receptive field or because separate groups of neurons encoding vertical and horizontal dimensions coexist within the fMRI voxel. Therefore, electrophysiology should complement human neuroimaging studies for a complete understanding of 3D space representation.
